# Histamine release from intestinal mast cells induced by staphylococcal enterotoxin A (SEA) evokes vomiting reflex in common marmoset

**DOI:** 10.1371/journal.ppat.1007803

**Published:** 2019-05-21

**Authors:** Hisaya K. Ono, Shouhei Hirose, Kouji Narita, Makoto Sugiyama, Krisana Asano, Dong-Liang Hu, Akio Nakane

**Affiliations:** 1 Department of Microbiology and Immunology, Hirosaki University Graduate School of Medicine, Hirosaki, Japan; 2 Department of Zoonoses, Kitasato University School of Veterinary Medicine, Towada, Aomori, Japan; 3 Department of Biopolymer and Health Science, Hirosaki University Graduate School of Medicine, Hirosaki, Japan; 4 Institute for Animal Experimentation, Hirosaki University Graduate School of Medicine, Hirosaki, Japan; 5 Department of Veterinary Anatomy, Kitasato University School of Veterinary Medicine, Towada, Japan; Geisel School of Medicine at Dartmouth, UNITED STATES

## Abstract

Staphylococcal enterotoxins (SEs) produced by *Staphylococcus aureus* are known as causative agents of emetic food poisoning. We previously demonstrated that SEA binds with submucosal mast cells and evokes mast cell degranulation in a small emetic house musk shrew model. Notably, primates have been recognized as the standard model for emetic assays and analysis of SE emetic activity. However, the mechanism involved in SEA-induced vomiting in primates has not yet been elucidated. In the present study, we established common marmosets as an emetic animal model. Common marmosets were administered classical SEs, including SEA, SEB and SEC, and exhibited multiple vomiting responses. However, a non-emetic staphylococcal superantigen, toxic shock syndrome toxin-1, did not induce emesis in these monkeys. These results indicated that the common marmoset is a useful animal model for assessing the emesis-inducing activity of SEs. Furthermore, histological analysis uncovered that SEA bound with submucosal mast cells and induced mast cell degranulation. Additionally, *ex vivo* and *in vivo* pharmacological results showed that SEA-induced histamine release plays a critical role in the vomiting response in common marmosets. The present results suggested that 5-hydroxytryptamine also plays an important role in the transmission of emetic stimulation on the afferent vagus nerve or central nervous system. We conclude that SEA induces histamine release from submucosal mast cells in the gastrointestinal tract and that histamine contributes to the SEA-induced vomiting reflex via the serotonergic nerve and/or other vagus nerve.

## Author summary

Staphylococcal enterotoxin A (SEA) is a bacterial toxin that has been recognized as a leading causative agent of staphylococcal food poisoning since 1930. The primary symptoms of staphylococcal food poisoning are nausea and emesis, which develop up to 1–6 h after ingestion of the causative foods contaminated by the bacteria. In the present study, we established the common marmoset as an emetic animal model and investigated the mechanisms of SEA-induced emesis in the primate model. Common marmosets that received SEA showed multiple emetic responses. We observed that SEA bound with submucosal mast cells in the intestinal tract and induced mast cell degranulation. Furthermore, SEA promoted histamine release from mast cells. We also demonstrated that histamine plays an important role in the SEA-induced emetic response in common marmosets. We conclude that SEA induces histamine release from submucosal mast cells in the intestinal tract and that the stimulation is transmitted from intestine to the brain via nerves, causing emesis. Our study provides a novel insight into functions of submucosal mast cells in the digestive tract.

## Introduction

Staphylococcal enterotoxins (SEs) produced by *Staphylococcus aureus* (*S*. *aureus*) have emetic activity and are causative agents of bacterial food poisoning. The primary symptoms of staphylococcal food poisoning include nausea, abdominal cramping and vomiting, which develop up to 1–6 h after ingestion of the causative foods contaminated with *S*. *aureus* [1]. In 1930, Dack *et al*. showed that staphylococcal food poisoning is not due to *S*. *aureus* cells, but caused by intoxication with SEs in the contaminated foods [2]. These toxins are also superantigens, which have the ability to activate a large amount of T cells [3]. These emetic and superantigenic activities make SEs a public health concern. Five major serological types of SEs (SEA to SEE), so-called “classical SEs”, have been characterized [3]. Notably, new types of SEs and SE-like toxins (SEG to SElV, SElX and SElY) have also been reported [3–10]. Although the mechanism of superantigenic activity and the amino acid residues in the active site of SEs have been clarified, the exact molecular and cellular mechanisms of their emetic activity still remain unclear. We have previously elucidated the mechanism of SEA-induced emesis using a small emetic animal model, house musk shrew (*Suncus murinus*) model. This study has revealed shown that SEA binds with submucosal mast cells and evokes mast cell degranulation [11]. We also have demonstrated that 5-hydroxytryptamine (5-HT) is a key molecule in SEA-induced emesis in house musk shrews [12]. Notably, the primates have been recognized as the standard model for detecting the emetic activity of SEs [13, 14]. Therefore, it is necessary to conduct experiments in a primate model. However, the high cost and limited availability of primates have led to a reduction in the investigation of SE-induced emesis using this model.

The common marmoset is a New World monkey that is small in size (average height of approximately 20 cm). This monkey can give birth twice a year. Hence, the handling and breeding of this animal are easier than those of other primates. Furthermore, its complete genome sequence and the transgenic marmosets have been reported [15]. This information makes this animal attractive for investigation of SE-induced emesis. In the present study, we established a new emetic animal model using the common marmoset and analyzed the emetic activity of SEA. In addition, we used histological and pharmacological techniques for common marmosets and their gastrointestinal (GI) tracts to clarify the mechanism of emesis induced by SEs.

## Results

### Establishment of an emetic model using common marmosets

In order to investigate the suitability of common marmosets for the emetic assay, monkeys were administered representative SEs SEA, SEB, SEC and SEI and the emetic response was observed for 5 h (Table 1). Five out of 8 common marmosets received 50-μg/kg of SEA and all 11 monkeys that had ingested 250-μg/kg of SEA exhibited vomiting responses from 45 to 202 min after administration. The vomiting responses occurred 4 to 9 times after 50 μg/kg of SEA treatment and 2 to 28 times after 250 μg/kg of SEA treatment. The emesis-inducing activity of SEB was comparable to that of SEA. Furthermore, ingestion of SEC induced emesis in 5 of 8 common marmosets. SEI and a new staphylococcal enterotoxin-like toxin named SEIY [10] exhibited an emetic response in common marmosets, although the frequency of the emetic response was lower than that of other SEs. By contrast, toxic shock syndrome toxin-1 (TSST-1), which is a non-emetic staphylococcal superantigen, showed no emetic response. The results indicated that common marmosets respond to the emetic activity of SEs.

**Table 1:** Emetic response induced by SEs in common marmoset.

Toxin	No. of monkey vomited/tested	Latency (min)	No. of emetic episodes
SEA 50 μg/kg	5/8	94, 103, 106, 108, 119	9, 7, 5, 5, 4
SEA 250 μg/kg	11/11	45, 79, 82, 97, 99, 100, 103, 108, 115, 134, 202	2, 5, 4, 28, 4, 11, 5, 13, 13, 15, 5
SEA 250 μg/kg i.v. *	2/6	187, 188	3, 12
SEB 250 μg/kg	7/8	139, 148, 152, 158, 166, 186, 208	10, 2, 6, 7, 3, 4, 10
SEC 250 μg/kg	5/8	61, 84, 103, 115, 137	7, 12, 3, 5, 7
SEI 250 μg/kg	2/8	105, 110	4, 3
SElY 250 μg/kg	3/8	60, 70, 89	12, 2, 4
TSST-1 250 μg/kg	0/8		
PBS	0/8		

* **i.v., intravenous administration**

**Fig 1:**
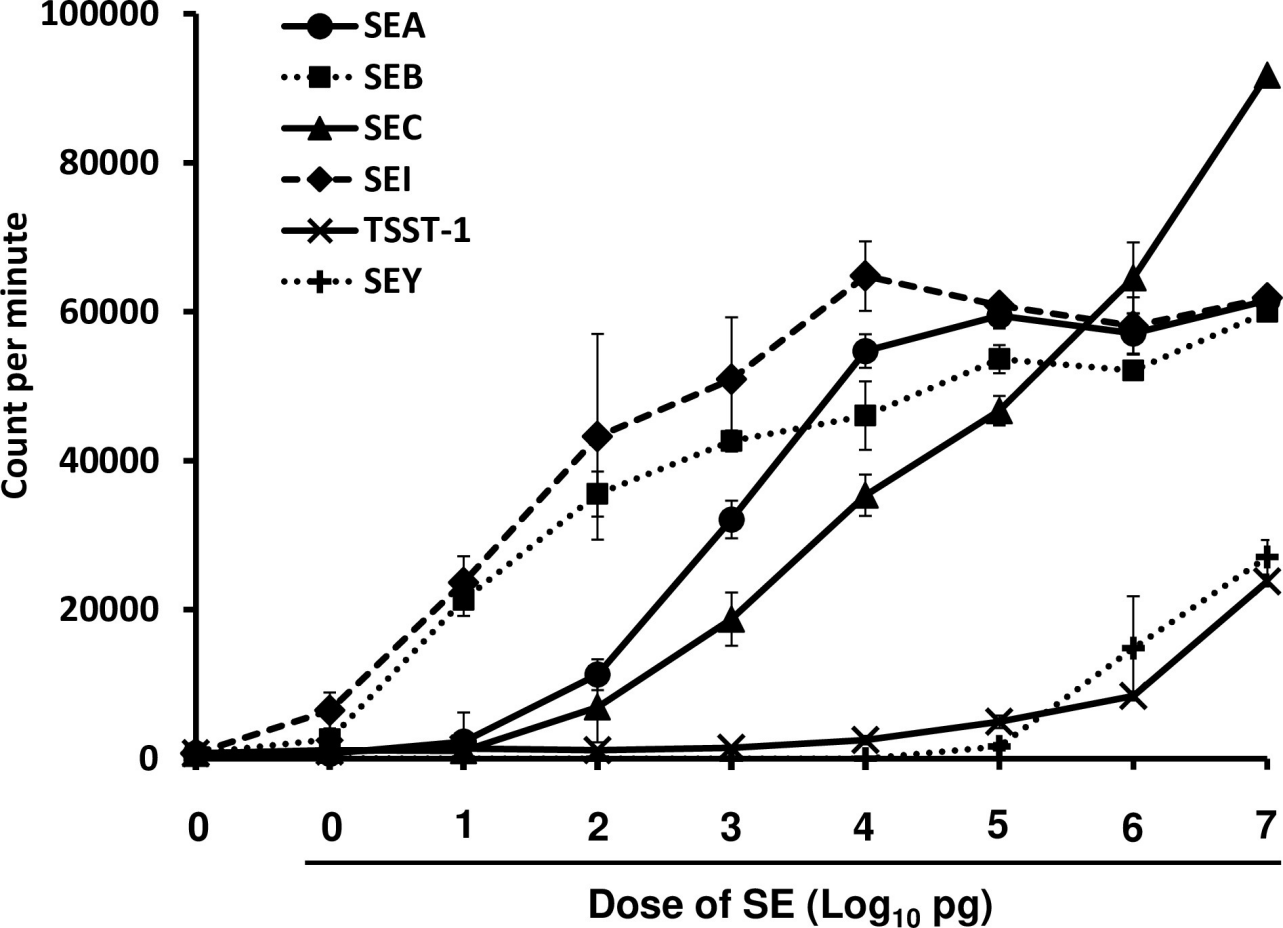
Superantigenic activity of SEs. PBMCs obtained from common marmosets were incubated with various concentrations of SEA, SEB, SEC, SEI, SEY or TSST-1 in 96-well round-bottomed tissue culture plates for 72 h and then uptake of [^3^H]-thymidine was measured. Data (in counts per min) are presented as the means ± SD of triplicate determinations.

SEs are also superantigens and have the ability to activate a large amount of T cells [3]. To clarify whether common marmosets respond to the superantigenic activity of SEs, the proliferation of common marmoset PBMCs stimulated with SEs, including SEA, SEB, SEC, SEI, SElY and TSST-1, was measured. The representative results from three experiments are shown in Fig 1. Over 1 pg/ml of SEB and SEI was able to induce PBMC proliferation. However, the minimum concentration of SEA and SEC required to induce PBMC proliferation was 100 pg/ml. To achieve substantial lymphocyte proliferation, these SEs required concentrations that were two orders of magnitude higher compared with SEB and SEI. The mitogenic activity of SElY and TSST-1 was low in comparison with that of other SEs. In the cases of SElY and TSST-1, the minimum concentration required to induce PBMC proliferation was 10 and 100 ng/ml, respectively. These results suggested that PBMCs of common marmosets responded to the superantigenic activity of SEs.

### SEA binds with submucosal cells in the GI tract

**Fig 2:**
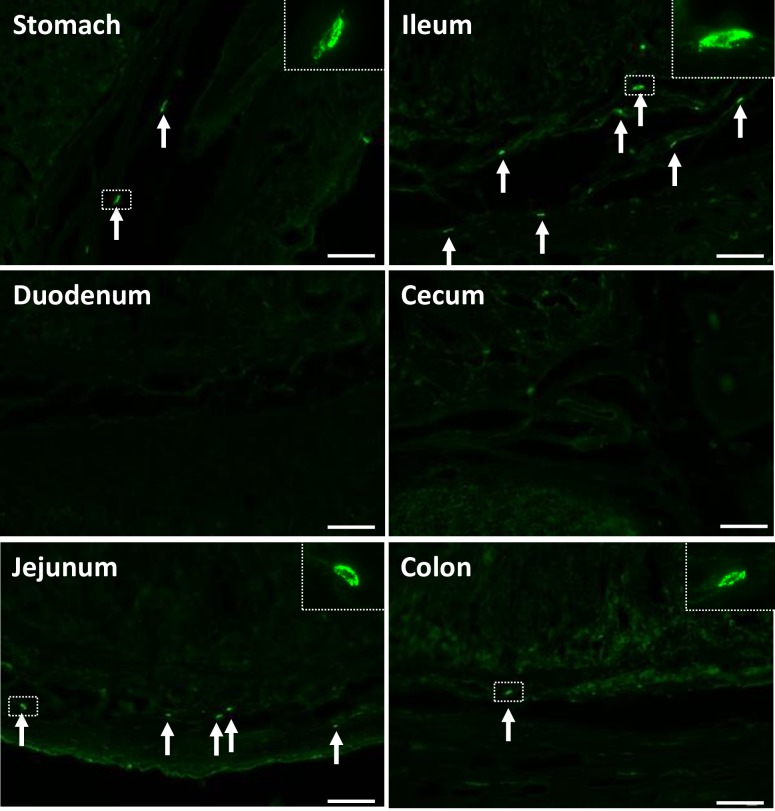
SEA binds with submucosal cells in the GI tract. GI tissues (stomach, duodenum, jejunum, ileum, cecum and colon) were obtained from a common marmoset. Frozen tissues were sectioned (10 μm) and incubated with SEA (1.0 μg/ml) for 60 min at room temperature. After washing the sections with PBST, the sections were stained for SEA using rabbit anti-SEA antibody and Alexa 488-conjugated goat anti-rabbit IgG. Magnification, x20. Each scale bar is equal to 50 μm.

We further explored the target cells of SEA in the GI tract of common marmosets. The GI tract was collected and each segment, including the stomach, duodenum, jejunum, ileum, cecum and colon, was incubated with 1 μg/ml of SEA for 60 min at room temperature. SEA-treated tissues were then processed for SEA immunostaining using anti-SEA polyclonal antibody. SEA-immunopositive cells were present in the submucosa of the jejunum and ileum (Fig 2). Only a few SEA-immunopositive cells were detected in stomach and colon. No SEA signal was detected in the mucosa or submucosa of the duodenum and cecum. Spindle-shaped morphology of SEA-positive cells was indicated in the submucosa of the jejunum and ileum. Notably, binding of SEA to enterochromaffin cells was not observed in the luminal epithelium of the GI tract. To confirm anti-SEA antibody signal specificity, SEA-treated GI tract sections were stained with non-immunized rabbit IgG primary antibody.

### SEA binds with submucosal mast cells

**Fig 3:**
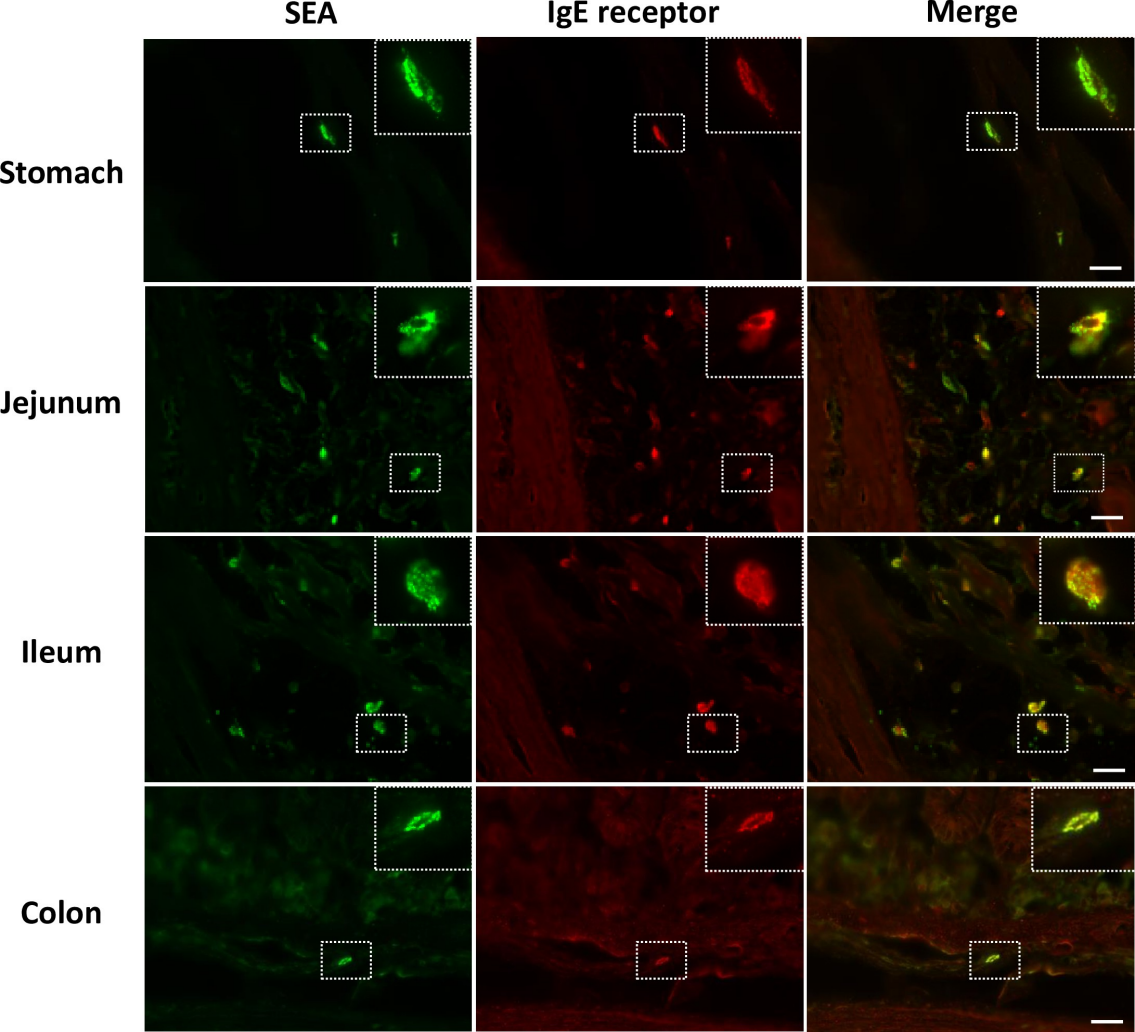
SEA binds with mast cells in the submucosa of the GI tract. Specimens of the intestinal tract were removed from a common marmoset. Frozen tissues were sectioned (10 μm) and incubated with SEA (1.0 μg/ml) for 60 min at room temperature. (A) GI sections were processed for double immunofluorescence staining using anti-SEA antibody (green) and anti-FcεRIα antibody (red), which detected the mast cell marker, IgE receptor. Each scale bar is equal to 20 μm.

**Fig 4:**
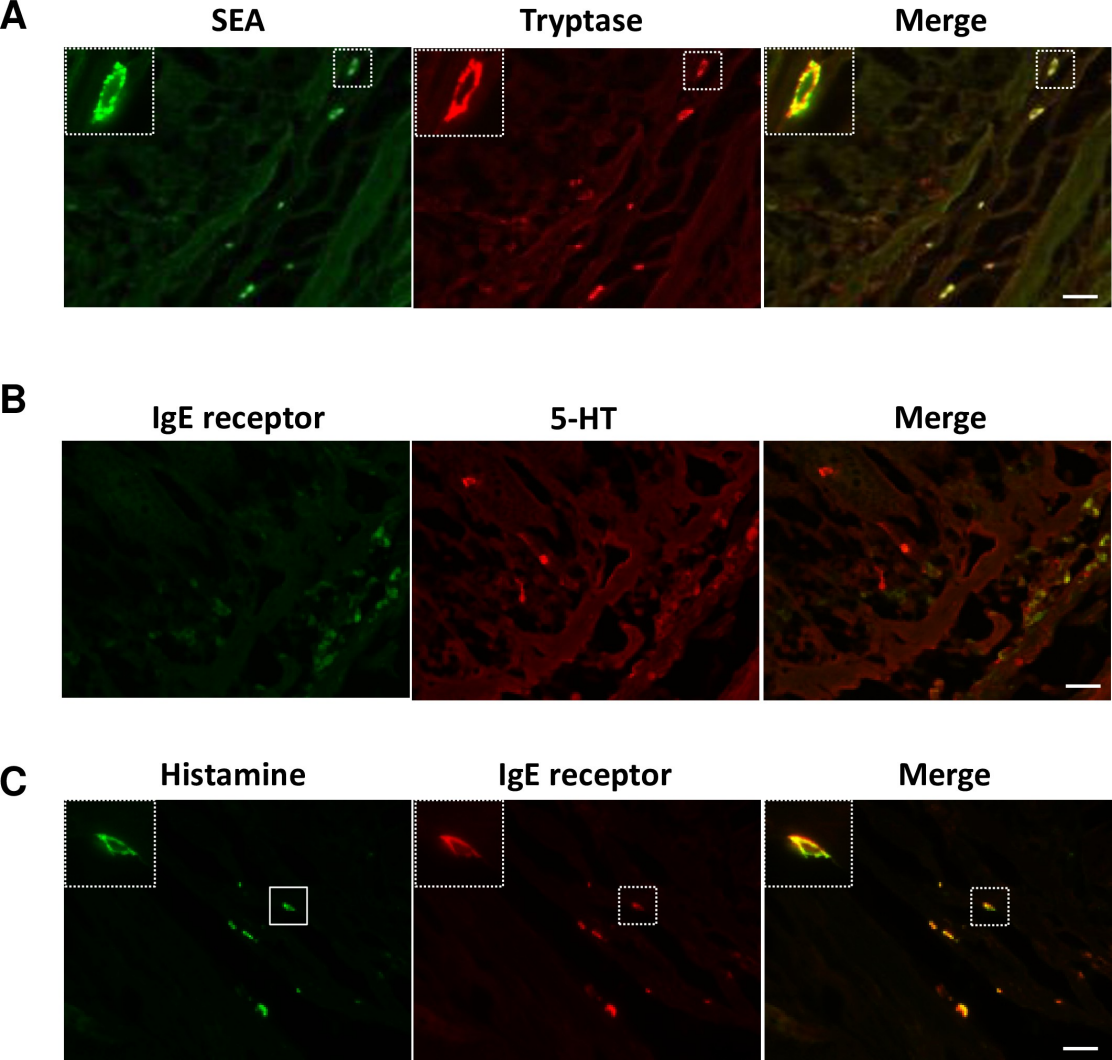
Characterization of SEA-binding mast cells. Specimens of the intestinal tract were removed from a common marmoset. Frozen tissues were sectioned (10 μm) and incubated with SEA (1.0 μg/ml) for 60 min at room temperature. (A) Ileal sections were processed for double immunofluorescence staining using anti-SEA antibody (green) and anti-tryptase mAb (red), which recognizes the mast cell marker, tryptase. (B) Ileal sections were processed for double immunofluorescence staining using anti-FcεRIα antibody (green) and anti-5-HT antibody (red). (C) Ileal sections were processed for double immunofluorescence staining using anti-histamine antibody (green) and anti-FcεRIα antibody (red). Each scale bar is equal to 20 μm.

Our previous study, which used house musk shrews, demonstrated that the target of SEA is submucosal mast cells [11]. In the present study, SEA-binding cells in the GI tract of common marmosets morphologically resembled connective tissue-type mast cells (Fig 2). Therefore, we investigated whether SEA binds with mast cells in the submucosal jejunum and ileum of common marmosets. Sections of GI tract were incubated with 1 μg/ml of SEA for 60 min at room temperature. The sections of SEA-treated jejunum and ileum were processed for double- immunofluorescence staining using anti-SEA antibody and antibody against FcεRIα, an IgE receptor that is known as a mast cell marker. As expected, SEA and FcεRIα signals were co-localized in the submucosal sections of the ileum (Fig 3). A similar result was observed in the jejunum sections (Fig 3), suggesting that SEA binds with mast cells. To further confirm whether the detected cells were mast cells, the ileal sections were stained with anti-tryptase monoclonal antibody (mAb), which recognizes tryptase on the mast cells. Double staining with antibodies against SEA and tryptase revealed that almost all of the SEA-binding cells were tryptase-immunopositive (Fig 4A). Additionally, SEA-immunopositive cells in the stomach and colon were also identified as mast cells (Fig 3). These results indicated that the target cells of SEA in the GI tract of common marmosets are most likely submucosal mast cells. In addition, SEA-treated ileal sections were stained with anti-5-HT mAb and anti-histamine mAb. As shown in Fig 4B and 4C, submucosal mast cells exhibited positive histamine signals, but not 5-HT.

### SEA induces degranulation in mast cells

**Fig 5:**
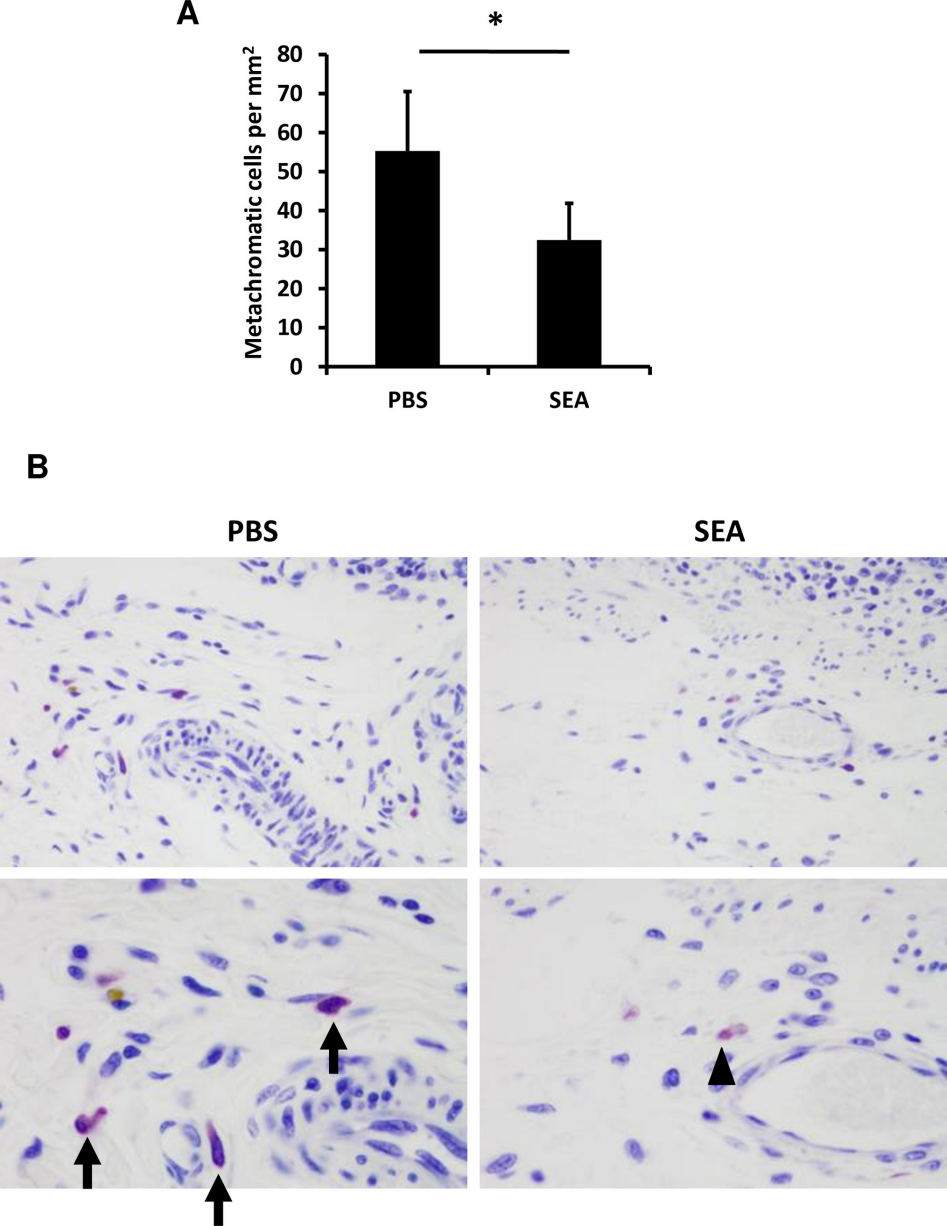
SEA induces degranulation of mast cells. Specimens of the intestinal tract were removed from a common marmoset at 2 h post-injection with 500 μg of SEA in the intestinal loop. (A) The number of metachromatic-staining cells in toluidine blue-stained jejunum sections was counted from 6 randomly selected areas. The number of submucosal metachromatic cells per square millimeter are presented as the mean ± SD, *P<0.05. (B) The jejunum sections in the intestine loop at 2 h after SEA or PBS injection were stained with toluidine blue. Mast cells are indicated as arrowheads in the SEA-injected loop and as arrows in the PBS-injected loop. The statistical analysis was performed using the Mann-Whitney U test.

Intact and degranulated mast cells are distinguishable by their metachromatic staining, particularly with toluidine blue [16, 17]. Thus, we further investigated whether the interaction between SEA and mast cells could induce degranulation by using toluidine blue staining. Specimens of the intestinal tract were removed from common marmosets at 2 h after injection with 500 μg of SEA in the intestinal loop. The results of toluidine blue staining showed that metachromatic mast cells, which are virtually identical to mast cells, were present in both the mucosa and submucosa in the GI tract. To estimate the extent of degranulation in the submucosal mast cells quantitatively, we compared the number of metachromatic-staining cells in toluidine blue-stained jejunum sections between SEA and phosphate-buffered saline (PBS) injection (Fig 5A). The results revealed that the number of metachromatic cells in the SEA-injected loop significantly was decreased in the jejunum submucosa compared with PBS-injected specimens (Fig 5A and 5B). These results suggested that mast cell degranulation occurs after SEA administration.

### SEA induces the release of histamine in GI tract

**Fig 6:**
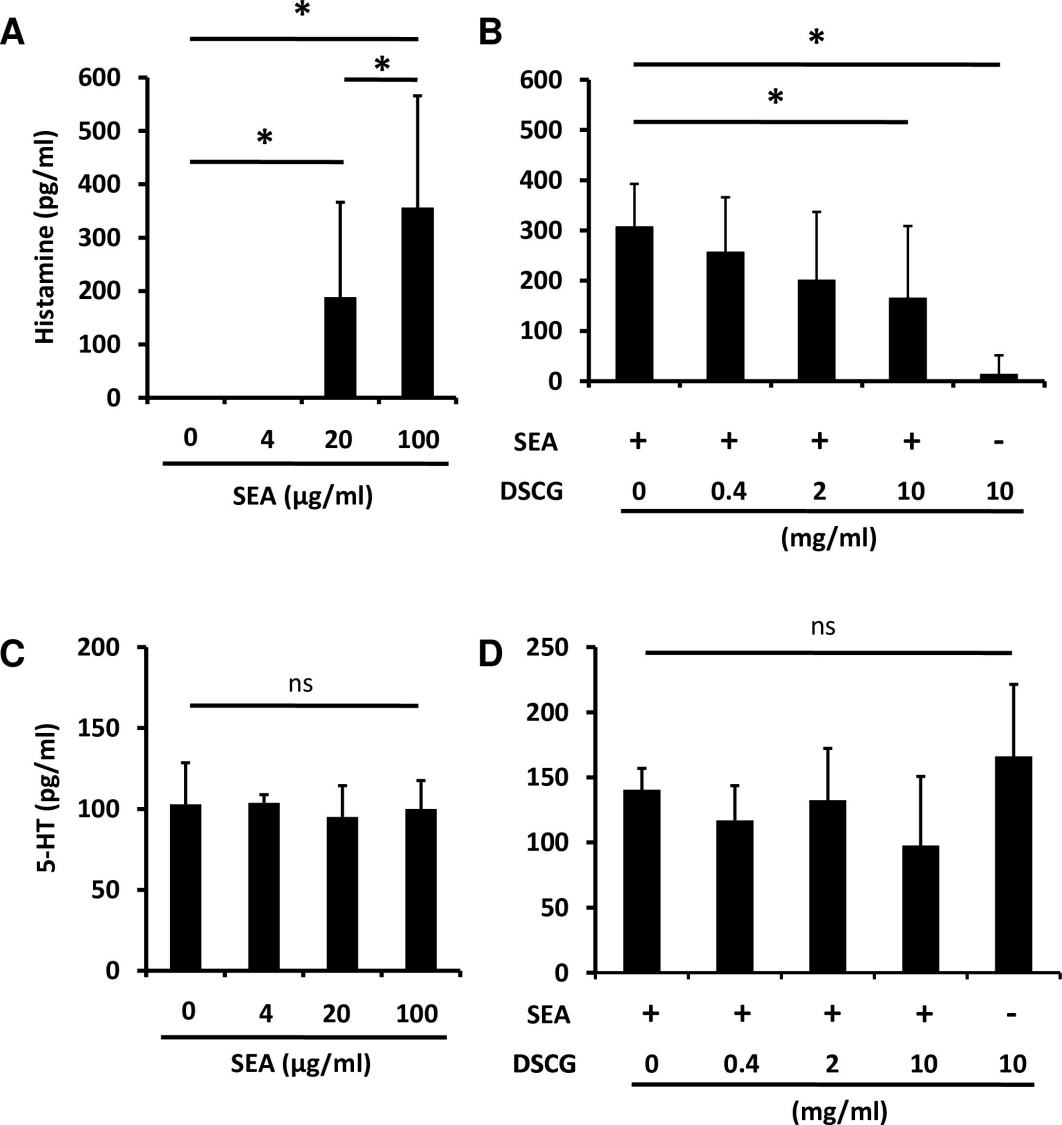
SEA induces the release of histamine from the GI tract. Sections of common marmoset jejunum were incubated with or without SEA, and histamine and serotonin released from the jejunum were measured. (A) Jejunal sections were incubated with various concentrations of SEA for 2 h at 37°C in a CO_2_ incubator and histamine in the culture supernatant fluid was measured using ELISA. (B) Jejunal sections were incubated with 100μg of SEA and various concentrations of DSCG for 2 h and histamine in the culture supernatant fluid was measured using ELISA. (C) Jejunal sections were incubated with various concentrations of SEA for 2 h and 5-HT in the culture supernatant fluid was measured using ELISA. (D) Jejunal sections were incubated with 100 μg of SEA and various concentrations of DSCG for 2 h and 5-HT in the culture supernatant fluid was measured using ELISA. Each bar represents the mean ± SD from 8 jejunal pieces. The statistical analysis was performed using ANOVA followed by Tukey’s post hoc test, *P<0.05.

We investigated whether chemical mediators, such as histamine, are released from SEA-induced mast cells. The sections of common marmoset jejunum were incubated with 0, 4, 20 or 100 μg/ml of SEA for 2 h at 37°C in a CO_2_ incubator. Following this, histamine and 5-HT release in the culture supernatant fluid was measured. Notably, SEA induced the release of histamine in the jejunal sections incubated with 20 and 100 μg/ml of SEA (Fig 6A). In order to confirm whether the observed SEA-induced histamine release caused by mast cell degranulation, the jejunum sections were incubated with mast cell stabilizer disodium cromoglycate (DSCG) during SEA-stimulation. DSCG suppressed SEA-induced histamine release in a dose-dependent manner (Fig 6B). However, 5-HT release from the jejunum was not affected by SEA stimulation or the addition of DSCG (Fig 6C and 6D), suggesting that 5-HT release from the jejunum is independent of SEA and mast cells. Taken together, these results strongly suggested that SEA induces degranulation and histamine release from the submucosal mast cells.

### Effect of mast cell stabilizer and H_1_ blocker on SEA-induced emesis

**Fig 7:**
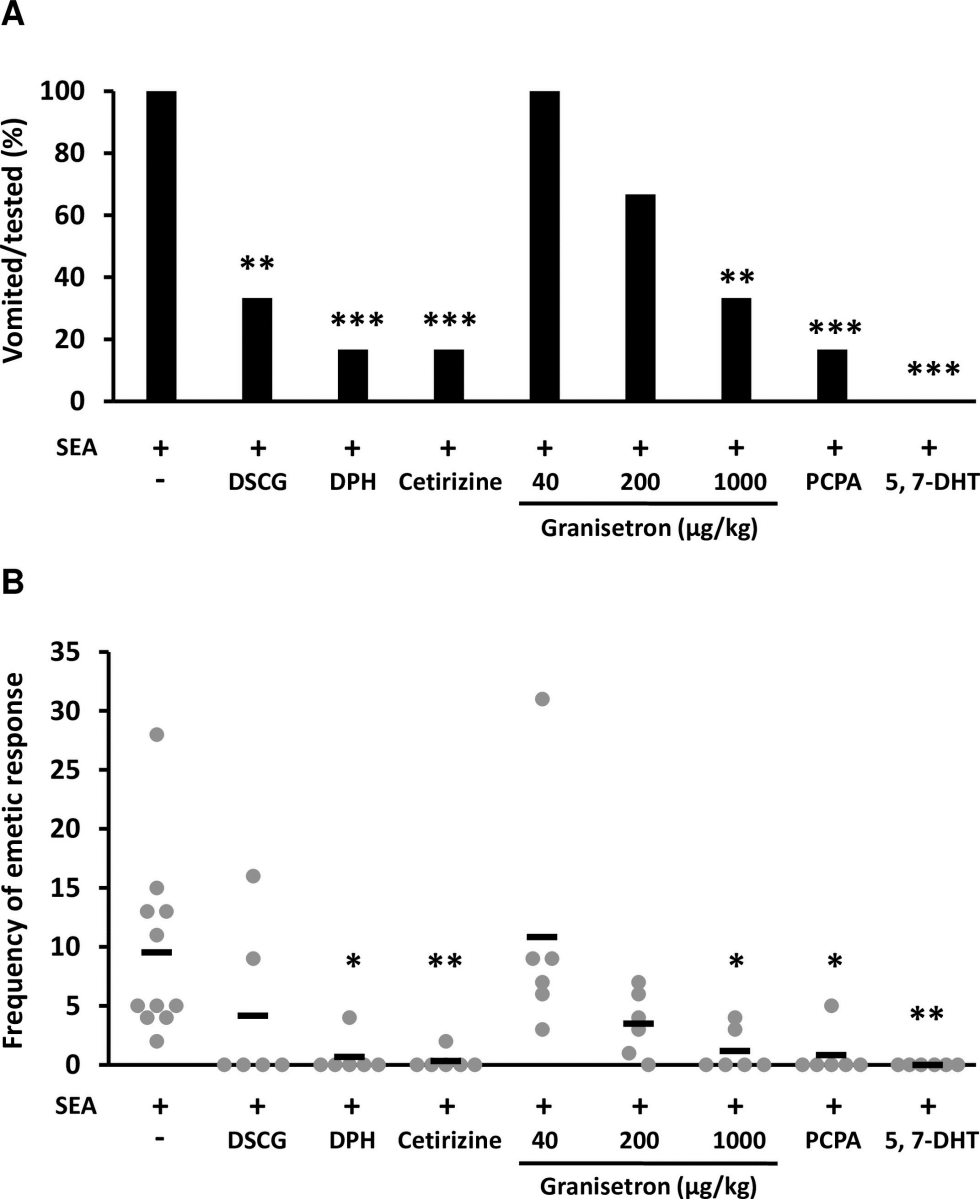
Pharmacological effects on SEA-induced emesis. Common marmosets were injected intraperitoneally with 200 mg/kg of DSCG (n = 6), 10 mg/kg of DPH (n = 6), 400 μg/kg of cetirizine (n = 6), 40 μg/kg of granisetron (n = 6), 200 μg/kg of granisetron (n = 6), 1000 μg/kg of granisetron (n = 6) or PBS (n = 11) and then administered 250 μg/kg of SEA 30 min later. To inhibit 5-HT synthesis, common marmosets were injected with PCPA (3500 mg/kg) over 2 consecutive days (n = 6) before SEA administration as described above. To deplete 5-HT in peripheral neurons without norepinephrine defects, common marmosets were pretreated with desipramine (25 mg/kg) for 60 min, then injected with 5 mg/kg of 5,7-DHT, followed by SEA administration as described above (n = 6). (A) The emetic response (percentage of vomited monkeys) and (B) its frequency were assessed as described in materials and methods. Each bar represents the mean ± SD. The statistical analysis was performed using Fisher’s exact test and Dunnett’s test, *P<0.05, **P<0.01, ***P<0.001.

To clarify whether SEA-induced submucosal mast cell degranulation is associated with emesis, the common marmosets were administered 250 μg/kg of SEA after DSCG injection. Four out of 6 common marmosets in the DSCG-treated group exhibited no vomiting response, and the number of emetic episodes was significantly decreased during observation in comparison with the group without DSCG pre-treatment (Fig 7A and 7B). Next, the effect of histamine H_1_ blockers, diphenhydramine (DPH) and cetirizine, on SEA-induced emesis was investigated. The vomiting response was suppressed in 5 out of 6 common marmosets in DPH-treated and cetirizine-treated groups, and the number of emetic episodes was significantly decreased in comparison with the control group receiving SEA alone (Fig 7A and 7B). These results indicated that SEA-induced degranulation and histamine release evokes emesis.

### 5-HT is involved in SEA-induced emesis in the central nervous system and peripheral nerves

To clarify whether 5-HT is involved in SE-induced emesis, the common marmosets were injected with granisetron (40 to 1000 μg/kg), a 5-HT_3_ receptor antagonist, and then administered 250 μg/kg of SEA. All marmosets exhibited vomiting responses following treatment with 40-μg/kg granisetron, whereas treatment with 200-μg/kg and 1000-μg/kg granisetron inhibited the vomiting response in a dose-dependent manner (Fig 7A and 7B). To confirm the effect of 5-HT on SEA-induced emesis, the monkeys were injected with *p*-chlorophenylalanine (PCPA), a 5-HT synthesis inhibitor, and then administered 250 μg/kg of SEA. Five of 6 marmosets in the PCPA-treated group exhibited no vomiting response and the number of emetic episodes was significantly decreased in comparison with the control group receiving SEA alone (Fig 7A and 7B). Furthermore, we investigated whether SEA-induced emesis is associated with serotonergic afferent nerve. The common marmosets were treated with serotonergic neurotoxin, 5,7-dihydroxytryptamine (5,7-DHT), and then administered 250 μg/kg of SEA. None of 6 marmosets in the 5,7-DHT-treated group exhibited an emetic response (Fig 7A and 7B). These results suggested that 5-HT is also involved in SEA-induced emesis and that 5-HT acts on the vagus nerve and/or central nervous system.

### Stimulation of emesis by SEA is transmitted via the vagus nerve

**Fig 8:**
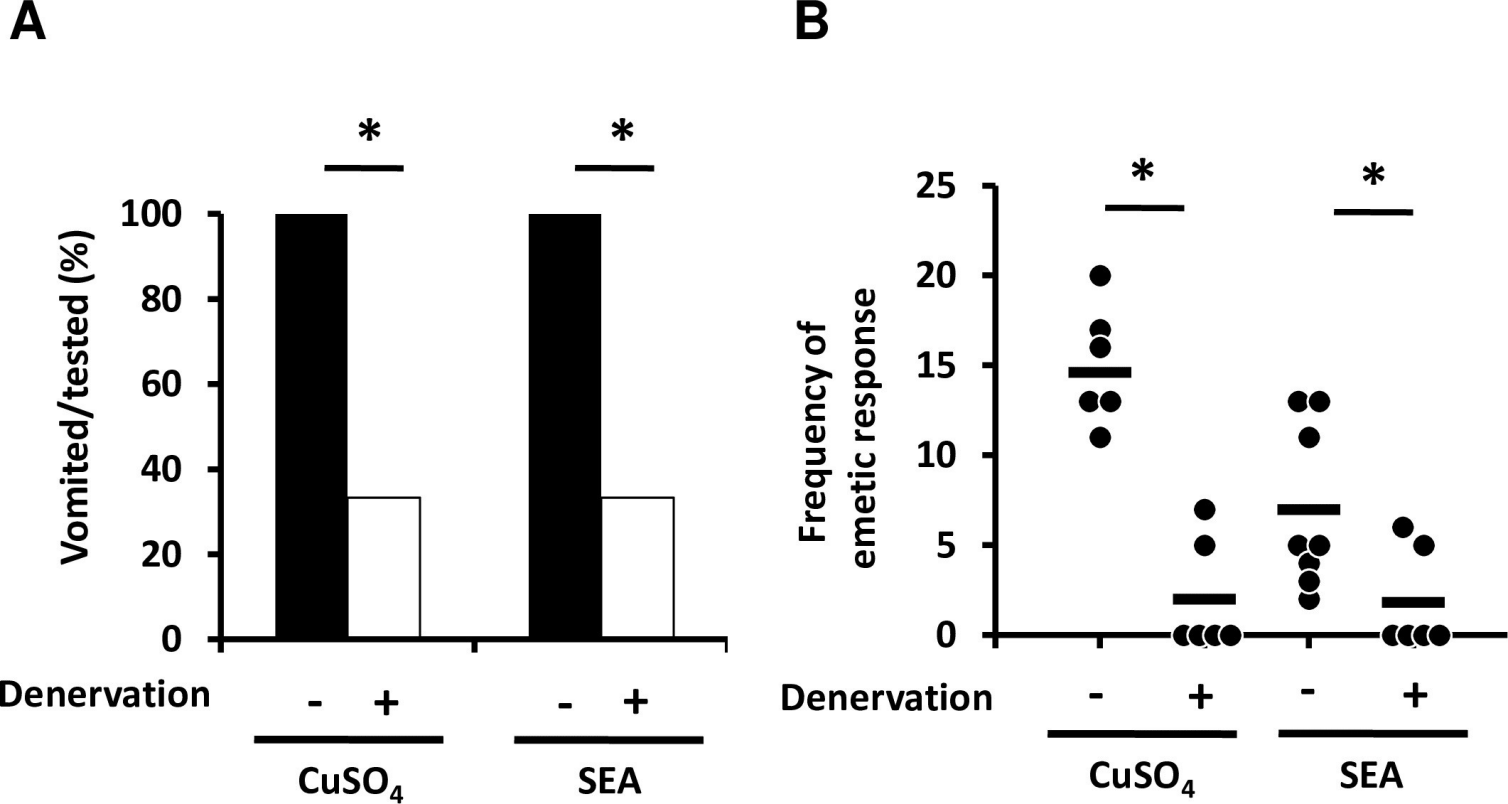
Stimulation of vomiting by SEA is transmitted via the vagus nerve. Vagotomized (n = 6) and sham operated (n = 6) common marmosets were administered 40 mg/kg of copper sulfate (left panel). Alternatively, vagotomized (n = 6) and sham-operated (n = 8) common marmosets were administered 250 μg/kg of SEA (right panel). (A) The emetic response (percentage of vomited monkeys) and (B) its frequency were assessed as described in materials and methods. Each bar represents the mean ± SD. The statistical analysis was performed using Fisher’s exact test and the Mann-Whitney U test, *P<0.05.

To clarify whether stimulation of vomiting by SEA is transmitted via the vagus nerve to the chemoreceptor trigger zone (CTZ) or vomit center, the common marmosets were vagotomized. To confirm whether the vagus nerves were substantially denervated, the monkeys were administered copper sulfate, a control substance that evokes vomiting via vagus nerves. Four out of 6 common marmosets in the copper sulfate-treated group and SEA-treated group exhibited no vomiting response, and the number of emetic episodes was significantly decreased in comparison with the non-vagotomized group (Fig 8A and 8B). Moreover, the difference of susceptibility to SEA-induced emesis between peroral and intravenous SEA administration was investigated to reveal whether SEA is able to act on CTZ directly. All 6 common marmosets exhibited vomiting responses between 45 and 134 min after peroral challenge with 250 μg/kg of SEA; however, 4 out of the 6 monkeys intravenously injected with the same dose of SEA exhibited no vomiting response and the number of emetic episodes was significantly decreased in comparison with the group challenged by peroral administration (Table 1). In addition, latency periods of emesis in the intravenously injected group were 187 min and 188 min, respectively. These latency periods were prolonged compared with those of peroral administration (Table 1). Taken together, these results suggested that SEA did not act on the CTZ directly. However, the findings did suggest that SEA-induced emesis was transmitted from GI tract to vomit center via the vagus nerve.

## Discussion

The mechanism of SEA-induced vomiting has not been fully elucidated in primates [2]. To investigate this mechanism, we established the common marmoset as an emetic animal model. In the present study, we showed that vomiting was induced in the common marmosets by orogastric intubation of SEs, including classical SEs and recently identified SEs (Table 1). However, none of common marmosets administered with TSST-1 or PBS exhibited an emetic reaction (Table 1). Notably, PBMCs of common marmosets were susceptible to the superantigenic activity of SEs (Fig 1). These results are consistent with responses of human and other emetic animal models [6, 11–13, 18–21]. Although TSST-1 has the highest toxicity to humans among the superantigens, the present study showed that the superantigenic activity of TSST-1 against common marmosets was markedly lower than that against humans. The results suggest the possibility that major histocompatibility complex and/or T cell receptors of common marmosets have low binding affinity to TSST-1. Furthermore, our results indicated that SEI displayed weak emetic activity but was strongly superantigenic. These SEI properties provide information that there is no complete relationship between emetic activity and superantigenic activity. This observation correlates with the data from Harris *et al*. and Schlievert *et al*. which have shown the separation of emesis from superantigenic activity [22, 23]. Our previous report indicated that 5 out of 10 cynomolgus monkeys administered 10 μg/kg of SEA, and 6 out of 7 cynomolgus monkeys that had ingested 100 μg/kg of SEA exhibited vomiting responses [21]. We also reported that house musk shrews could be used as a small emetic animal model and that shrews were highly sensitive to SEA in the initial report (32 μg/kg, orogastric administration) [19]. However, our recent study showed that house musk shrews required a larger amount of SEA (1.4–2.0 mg/kg of body weight) to promote emesis, indicating a decreased susceptibility of shrews to SEA [24]. Therefore, it was assumed that the marmosets used in the present study showed almost the same sensitivity to SEA as cynomolgus monkeys. As the body weight of common marmosets was markedly low (approximately 0.3 kg), a lower amount of toxins was required compared with cynomolgus monkeys (1.4–2.0 kg). Furthermore, the complete genome sequences and transgenic marmosets have been reported [25, 26]. Hence, the common marmoset may be a useful animal model for detection and analysis of SEs.

In 2004, the *International Nomenclature Committee for Staphylococcal Superantigens* (INCSS) recommended that only toxins that could induce vomiting after oral administration in primates are termed SEs [14]. Our results indicate that SElY has emetic activity in primates (Table 1). Therefore, in the present study it was proposed that SElY should be renamed SEY, according to INCSS recommendations.

Our previous report showed that SEA binds to submucosal mast cells in the GI tract in house musk shrews and can induce submucosal mast cell degranulation, as well as 5-HT release [11]. To clarify the mechanism of SEA-induced emesis in common marmosets, we used histological and pharmacological techniques in the present study. SEA was indicated to bind with submucosal cells in the GI tract, specifically in the stomach, jejunum, ileum and colon of common marmosets (Fig 2). We identified and characterized cells as submucosal mast cells according to the positive signals of IgE receptor, tryptase and histamine (Figs 3 and 4).

As indicated in Fig 5, SEA induced submucosal mast cell degranulation in the jejunum 2 h after SEA injection. Interestingly, SEA induced histamine release but not 5-HT release in the *ex vivo* experiments, and mast cell stabilizer reduced this histamine release (Fig 6). Furthermore, mast cell stabilizer and histamine H_1_ blockers reduced SEA-induced emesis induced in common marmosets (Fig 7). In brief, the degranulation of submucosal mast cells was promoted by SEA, and the inhibition of submucosal mast cell degranulation prevented SEA-induced emesis. These results suggested that submucosal mast cell degranulation is important in SEA-induced emetic responses.

In this study, we demonstrated for the first time that histamine release has a pivotal role in the emetic response in the GI tract (Figs 6 and 7). Conventionally, 5-HT from enterochromaffin cells in the GI mucosa has been considered to be an important mediator for anticancer drugs, chemical substances and vomiting due to food poisoning [27, 28]. Histamine is a key molecule for transmitting stimuli from the inner ear to the brain during vomiting due to motion sickness and also plays a role in the GI tract with regard to food allergies and histamine fish poisoning [29–31]. However, it has not been considered to be involved in vomiting associated with bacterial food poisoning [29, 32]. Mast cells in rodents are classified into mucosal mast cells (MMCs) and connective tissue mast cells [33, 34]. Human mast cells are divided into two major groups: MC_T_ (containing tryptase) and MC_TC_ (containing tryptase and chymase) [35, 36]. In the human GI tract, MMCs are MC_T_, whereas submucosal mast cells mostly exhibit MC_TC_ properties [36]. MMCs have been known to be important cells in allergy development [37]. However, a few reports demonstrated that submucosal mast cells of the GI tract are involved in diseases, and their function is still unknown in various respects [32, 34]. The present study indicated that submucosal mast cells were involved in the emetic response and that SEA specifically affects submucosal mast cells in the GI tract. This study provides a novel insight into the functions of submucosal mast cells in the GI tract.

In summary, the results of the present study have shown that the common marmoset is a useful animal for emetic assays and that submucosal mast cells and histamine play critical roles in SEA-induced emesis in common marmosets. Furthermore, 5-HT plays an important role in the transmission of emetic stimulation on the afferent vagus nerve or central nervous system [38]. Taken together, our results suggest that the binding of SEA with mast cells induced histamine release, which acts against the serotonergic nerve and/or other vagus nerve. Furthermore, it was indicated that stimulation could be transmitted to the vomiting center, causing a vomiting reflex. These findings provide a novel function for mast cells and the vomiting pathway, which are considered to be essential in cell biology and neuroscience.

## Materials and methods

### Ethical statement

This study was conducted in accordance with the Declaration of Helsinki. All animal experiments were approved by the Animal Research Ethics Committee of Hirosaki University Graduate School of Medicine (permit number M10037), and followed the Guidelines for Animal Experimentation, Hirosaki University. The Hirosaki University guidelines are in accordance with the guidelines for proper conduct of animal experiments determined by Ministry of the Environment *Standard relating to the Care and Keeping and Reducing Pain of Laboratory Animals* (Notice of the Ministry of the Environment No. 88 of 2006) and American Veterinary Medical Association (AVMA) guidelines for the euthanasia of animals: 2013 edition. Common marmosets (*Callithrix jacchus*) were purchased from CLEA Japan, Inc. (Tokyo, Japan). Common marmosets were housed at 26–30°C in a room lit for 12 h (from 8:00 a.m. to 8:00 p.m). The monkeys had *ad libitum* access to tap water and food pellets (CMS-1M, CLEA Japan, Inc.). One or two monkeys were housed per cage (420x620x670 mm). Daily care was provided by the same staff, and the veterinary staff took care of the monkeys in case of any health problems. Eleven sexually mature monkeys (aged 2–4 years old and weighing 280–360 g) were used in each experiment. To reduce the number of common marmosets used in this study, each monkey was repeatedly administered different types of toxins. The monkeys were then used for experiments with inhibitors. For tissue sample preparation, common marmosets were euthanized by exsanguination under fully anesthetization using a mixture of medetomizine, midazolam and butorphanol and/or inhaled isoflurane.

### Preparation of toxins

Cloning and preparation of recombinant SEA, SEB, SEC, SEI, SElY and TSST-1 were performed as described previously [10, 20, 21]. Briefly, the toxin genes from *S*. *aureus* isolates were amplified by PCR and the PCR products were digested with *Bam*HI and *Eco*RI or *Sal*I. The fragment of the genes was then cloned into pGEX6P-1, a glutathione S-transferase (GST) fusion expression vector. Expression and purification of the GST-fused recombinant proteins and the cleavage and removal of the GST tag from recombinant SE proteins were performed by the methods described previously [10, 21]. Recombinant toxins were treated with a ProteoSpin Endotoxin Removal mini kit (Norgen Biotek Corp., Thorold, ON, Canada).

### Assay of emetic activity

The emetic activity of SEs in common marmosets was estimated using Bergdoll’s monkey feeding assay with some modifications [13]. At 16 h after food deprivation, common marmosets were anesthetized by an intramuscular injection with a mixture of medetomizine (80 μg/kg; ZENOAQ, Fukushima, Japan) and midazolam (400 μg/kg; SANDOZ, Tokyo, Japan). SEA, SEB, SEC, SEI, SElY or TSST-1 was dissolved in 1.5 ml of sterile PBS and fed to common marmosets at a dose of 50 or 250 μg/kg by orogastric intubation. Alternatively, SEA was dissolved in 150 μl of sterile PBS and administered intravenously at a dose of 250 μg/kg into the femoral vein. Following this, the monkeys were intramuscularly administered atipamezole (320 μg/kg, ZENOAQ) for rapid recovery. The emetic responses were recorded using a video camera for 5 h after oral administration of each toxin. The number of emetic responses, the latency period of the first emetic response and behavioral changes were evaluated. In order to avoid the undesirable influences by repeated administration of the same SE, we did not use the same SE in an individual marmoset with the exception of SEA when the responses to various doses were compared.

### Assays of superantigenic activity

Mitogenic activity of SEs was determined using PBMCs of common marmosets. Blood from 3 healthy monkeys was processed using Ficoll-Paque PLUS (GE Healthcare Japan, Tokyo, Japan) density-gradient centrifugation. PBMCs were resuspended in RPMI-1640 medium (Nissui Pharmaceutical Co. Ltd., Tokyo, Japan) containing 10% fetal bovine serum (FBS, Nichirei Bioscience, Tokyo, Japan) and penicillin/streptomycin. The PBMCs were then incubated for 72 h in 96-well round-bottomed tissue culture plates (AGC Techno Glass Co., Shizuoka, Japan) with different concentrations of SEA, SEB, SEC, SEI, SEY or TSST-1 and then assayed for the uptake of [^3^H]-thymidine (PerkinElmer Japan Co., Ltd., Kanagawa, Japan). Data (in counts per min) were presented as the mean μ standard deviation (SD) of triplicate determinations, as previously described [39].

### Tissue preparation for immunostaining

Common marmosets were euthanized by exsanguination under deep anesthesia with a mixture of medetomizine (120 μg/kg), midazolam (600 μg/kg) and butorphanol (600 μg/kg; Meiji Seika Pharma Co., Ltd., Tokyo, Japan). Specimens of the GI tract were removed, washed with ice-cold PBS and fixed overnight with Mildform 10NM (Wako Pure Chemical Industries, Ltd., Osaka, Japan). Subsequently, tissue samples were soaked in 30% sucrose in PBS overnight at 40°C and frozen in Optimal Cutting Temperature medium (Sakura Finetek, Tokyo, Japan) at -80°C.

### Immunofluorescence analysis

Cryostat sections (10 μm) were processed for immunofluorescence staining with single or double labeling. The sections were washed in PBS and incubated with 2% normal goat serum (Jackson ImmunoResearch, West Grove, PA, USA) in PBS/0.05% Tween 20 (PBST) for 30 min at room temperature. Afterwards, the sections were rinsed in PBST and incubated with SEA (1.0 μg/ml) for 60 min at room temperature. After washing the sections with PBST, rabbit polyclonal anti-SEA antibody was used to detect SEA in the tissue. This rabbit polyclonal anti-SEA antibody (0.2 μg/ml) was obtained from sera of SEA-immunized rabbits and purified by affinity chromatography using a HiTrap kit (GE Healthcare Japan), as previously reported [11]. Rat anti-5-HT mAb (1:200; EMD Millipore, Billerica, MA, USA), rabbit polyclonal anti-histamine antibody (1:200; EMD Millipore), mouse anti-tryptase mAb (1:1000; Agilent Technologies, Inc., Santa Clara, CA, USA) and mouse anti-FcεRIα mAb (clone CRA1 1:1000; BioAcademia, Osaka, Japan) were also used as primary antibodies. Incubation of the sections with primary antibodies was carried out at 40°C overnight. Then, the sections were rinsed in PBST and incubated with the following secondary antibodies: Alexa 488-conjugated donkey anti-rabbit IgG, Alexa 568-conjugated donkey anti-rat IgG and/or Alexa 568-conjugated donkey anti-mouse IgG (1:1000; Life Technologies Japan Ltd., Tokyo, Japan) for 60 min at room temperature. All of these antibodies were diluted in Can Get Signal Immunostain Solution A (Toyobo Life Science, Osaka, Japan). After rinsing with PBST, the sections were coverslipped with Prolong Gold antifade reagent (Life Technologies Japan) and examined using a fluorescence microscope (BZ-X700; Keyence, Osaka, Japan).

### Metachromatic analysis

Common marmosets were anesthetized by an intramuscular injection with a mixture of medetomizine (80 μg/kg) and midazolam (400 μg/kg) at 16 h after food deprivation. Anesthesia during surgical treatment was maintained using 1.0% isoflurane, an inhalational anesthetic. The small intestine was ligated in separate loops of 4 to 5 cm in length [40]. Intestinal loops were injected 0.5 ml PBS (with or without 500 μg of SEA) using a 27-gauge needle. Specimens of the intestinal loop were removed from common marmosets at 2 h after SEA injection. The specimens were fixed for 2 h with Methanol Carnoy’s solution (60% methanol, 30% chloroform and 10% glacial acetic acid) [41, 42]. Following this, tissue samples were paraffin-embedded according to the standard procedure. Paraffin-embedded tissue samples were cut in 4- μm-thick sections. Sections were deparaffinized and stained with 0.1% toluidine blue solution. The numbers of submucosal metachromatic cells in the intestinal tract were presented as the mean ± SD of *n* observations per square millimeter. The statistical analysis was performed using the Mann-Whitney U test.

### Measurement of histamine and 5-HT released from marmoset intestine

For *ex vivo* culture of the intestine, specimens of the jejunal tract were removed from common marmosets and transferred in RPMI-1640 medium supplemented with 10% FBS and penicillin/streptomycin. The jejunal pieces were incubated with SEA at different concentrations or with 100 μg SEA and different concentrations of DSCG for 2 h at 37°C in 5% CO_2_ incubator. Histamine and 5-HT in the culture supernatant fluid were measured using a Histamine ELISA kit (Enzo Life Sciences, Inc., Farmingdale, NY, USA) and a Serotonin ELISA kit (DRG International Inc., Springfield, NJ, USA), respectively. Statistical analysis was performed using ANOVA followed by Tukey’s post hoc test

### Mast cell stabilizer drug and mediator antagonists

Common marmosets received an intraperitoneal injection of the mast cell stabilizer drug, DSCG (200 mg/kg of weight; Wako Chemicals GmbH, Neuss, Germany), 1st generation histamine H_1_ receptor antagonist DPH (10 mg/kg of weight; Sigma-Aldrich; Merck KGaA, Darmstadt, Germany), 2nd generation histamine H_1_ receptor antagonist cetirizine (400 μg/kg of weight; Sigma-Aldrich; Merck KGaA) or 5-HT_3_ receptor antagonist granisetron (Sigma-Aldrich; Merck KGaA), diluted with saline. A total of 30 min later, the animals received SEA (250 μg/kg) diluted with PBS by orogastric intubation. Following this, the emetic response in these monkeys was observed for 5 h. The number of vomiting marmosets, the number of emetic episodes (frequency of vomiting) and any behavioral changes during the 5-h observation period were monitored using a video camera recorder. Six marmosets were used in each experiment. The statistical analysis was performed using Fisher’s exact test and Dunnett’s test.

### Inhibition of 5-HT synthesis

The 5-HT synthesis inhibitor PCPA (Sigma-Aldrich; Merck KGaA) was suspended in 4% gum arabic (Wako Chemicals GmbH) solution. Common marmosets were intraperitoneally injected with PCPA (3500 mg/kg of weight) over 2 consecutive days and administrated SEA (250 μg/kg of weight) by orogastric intubation 30 min after PCPA injection. Then, the emetic response in these monkeys was observed as described above.

### The effect of serotonergic neurotoxin 5,7-DHT on SEA-induced emesis

Common marmosets were injected intraperitoneally with 5,7-DHT (5 mg/kg of weight) to deplete 5-HT in peripheral neurons. To inhibit the depletion of norepinephrine, these monkeys were pretreated with desipramine (25 mg/kg of weight) via an intraperitoneal injection for 60 min. Animals received SEA (250 μg/kg) diluted with PBS via orogastric intubation. Then, the emetic response in these monkeys was observed as described above.

### Vagal denervation

Common marmosets were anesthetized using an intramuscular injection of medetomizine (60 μg/kg of weight), midazolam (300 μg/kg of weight) and butorphanol (300 μg/kg of weight). Surgical denervation was performed by cutting the vagus nerve at the GI level. To confirm whether denervation was successful, the animals were orally administered copper sulfate (40 mg/kg of weight) and the reduction of the emetic response in these monkeys was observed. At the same time, the vagotomized common marmosets were administered 250 μg/kg of SEA by orogastric intubation. The emetic response in these monkeys was observed as described above.

### Statistical analysis

Statistical tests undertaken for individual experiments are mentioned in the figure legends. *P*<0.05 was considered to indicate a statistically significant difference.
